# When Rarity Hits Twice: Hemophagocytic Lymphohistiocytosis in Kabuki Syndrome—A Case Report From Palestine

**DOI:** 10.1002/ccr3.72273

**Published:** 2026-03-11

**Authors:** Lilyan Jarrar, Raya Fuqha, Ahmad Mashni, Mohammad Hamdan, Sameer Alomari, Mohammad Ataya, Yazan Abed, Laith Abed

**Affiliations:** ^1^ Faculty of Medicine and Health Sciences An‐Najah National University Nablus Palestine; ^2^ Department of Pediatrics, Allergy and Immunology Unit Governmental Hospital, Palestinian Ministry of Health Nablus Palestine; ^3^ H‐Clinic Hospital Ramallah Palestine; ^4^ Jenin Governmental Hospital Jenin Palestine; ^5^ Al‐Maqased Hospital Jerusalem Palestine; ^6^ Jenin Governmental Hospital Jenin Palestine

**Keywords:** case report, genetics, hemophagocytic lymphohistiocytosis, kabuki syndrome

## Abstract

Clinicians should maintain a high index of suspicion for Hemophagocytic Lymphohistiocytosis in Kabuki syndrome patients who present with persistent fever, cytopenias, and organomegaly. Prompt diagnosis and multidisciplinary management are essential to improve outcomes in this rare but potentially fatal complication.

## 1 Introduction

Kabuki syndrome is a rare congenital disorder characterized by distinctive facial features, skeletal anomalies, and multisystem involvement [1]. Increasing evidence suggests that affected patients may also develop immune dysregulation, including autoimmune manifestations and hematological abnormalities. Hemophagocytic lymphohistiocytosis (HLH) is a life‐threatening hyperinflammatory syndrome caused by excessive immune activation, which can lead to multiorgan failure, tissue damage, and death [2]. HLH is classified into primary (familial) forms, resulting from inherited defects in cytotoxic lymphocyte function, and secondary forms, which are acquired and triggered by infections, autoimmune diseases, malignancies, or metabolic conditions [3]. In the setting of immune dysregulation and autoimmunity, secondary HLH may present as macrophage activation syndrome (MAS), a related hyperinflammatory condition with overlapping diagnostic criteria [4]. Here, we report a case of a child with Kabuki syndrome who developed overlapping features of HLH and autoimmunity.

## 2 Case Presentation/Examination

A 5‐year‐old boy, known case of Kabuki syndrome, was referred to our hospital with a history of recurrent sinopulmonary infections, bilateral sensorineural hearing loss, recurrent pneumonia, cervical lymphadenopathy, hypospadias, 2 weeks of fever (~39 degrees) that did not respond to antipyretics and empiric antibiotics, dental abscess, and aphthous ulcers. He had multiple hospitalizations and blood transfusions without any reported diagnosis. He has a global developmental delay. His weight is 19 kg (27th percentile), height 103 cm (below 3rd percentile), and body mass index (BMI) 17.91 (93rd percentile).

On physical examination, he appears pale and feverish (temperature 39.5 degrees), with a blood pressure of 104/54 mmHg. He exhibited cushioned and dysmorphic features that are consistent with Kabuki syndrome, including a widened nasal bridge, protruding ears, flattened upper lip, and hypoplasia of the medial cheeks. Abdominal examination revealed hepatosplenomegaly. Because of the persistence of cervical lymph nodes, a chest X‐ray was ordered, which showed consolidation in the left lower lung and mediastinum widening. An abdominal ultrasound confirmed the hepatomegaly and splenomegaly with 3 splenules. Abdominal CT showed a number of retroperitoneal lymph nodes and a right ectopic kidney. Echocardiography showed normal systolic function without hypertrophy. He was stabilized with 300 mL of packed red blood cells (PRBCs) and given wide‐spectrum antibiotics.

During his admission, his blood pressure was 159/117 mmHg (right arm), 115/91 mmHg (left arm), and both > 99th percentile. Serial blood pressure measurements confirmed hypertension, and antihypertension management was started.

## 3 Methods

### 3.1 Differential Diagnosis

Differentials such as CHARGE, Noonan, DiGeorge syndromes, primary immunodeficiency, sepsis, and malignancy were considered. Whole‐exome sequencing confirmed a KMT2D mutation consistent with Kabuki syndrome. Bone marrow showed only pancytopenia without malignancy, and an infectious workup, including CMV and EBV, was negative. With six of eight HLH‐2004 criteria fulfilled, the final diagnosis was Kabuki syndrome complicated by secondary HLH.

### 3.2 Investigations

Laboratory findings included pancytopenia (Table 1) and markedly elevated ferritin. The reported hemoglobin level (2.5 g/dL) and reticulocyte count (14%) were obtained prior to blood transfusion.

**TABLE 1:** Laboratory test results on admission.

Test	Result	Reference range
Hemoglobin	2.5	11.5–13.5 g/dL
ANC	0.04	1.5–8.0 × 10^9^ cells/L
Platelets	10,000	150–450 × 10^9^/L
Reticulocytes	14%	0.5%–1.5% of RBCs
LDH	1012	100–330 U/L
ESR	100	0–10 mm/hour
Ferritin	1440	7–140 ng/mL
Triglycerides	200	< 100 mg/dL
LDL	250	< 110 mg/dL
ANA	++4	Negative
C‐ANCA	Negative	Negative
P‐ANCA	Negative	Negative

Autoimmune markers included positive ANA, lupus anticoagulant, and anti‐neutrophil antibodies. The immunoglobulin profile showed skewed IgG, IgA, and IgM levels.

Genetic and bone marrow evaluation showed pancytopenia without malignancy or clear hemophagocytosis. Genetic testing confirmed a mutation consistent with Kabuki syndrome.

The sweat chloride test was negative, and kidney function tests were normal. A pediatric hematologist was consulted regard the patient's results, and suggested an autoimmune Lupus like disease and APLA with secondary HLH according to the HLH‐2004 diagnostic criteria, he got 6/8 (fever, cytopenias, hypertriglyceridemia, splenomegaly, elevated CD25 levels, and ferritin levels) in a child with Kabuki syndrome that is known for various autoimmune phenomenon. Natural killer cell activity and fibrinogen levels were not available at our center and, therefore, were not included in the diagnostic evaluation.

### 3.3 Management

The patient was managed with prednisone 24 mg/day, cyclosporine 30 mg/kg/day, folic acid 5 mg/day, Amlodipine 2.5 mg × 2/day, and hydrochlorothiazide 12.5 mg × 2/day. He received intravenous immunoglobulin (IVIG 2 g/kg) and required blood transfusions. At follow‐up, over the course of treatment, his clinical condition improved with resolution of fever, stabilization of blood counts, and normalization of blood pressure. He was also consulted for a low salt and fat diet and routine blood pressure measurements.

## 4 Discussion

Kabuki syndrome (KS) is a multisystem neurocristopathy caused predominantly by KMT2D and less often KDM6A variants, with downstream epigenetic effects on immune development and function [1, 2, 5]. Beyond the typical craniofacial and neurodevelopmental features, KS frequently exhibits clinically significant immune dysregulation, hypogammaglobulinemia/CVID‐like phenotypes, reduced class‐switched memory B cells, impaired T‐cell help, and variable NK‐cell dysfunction, contributing to recurrent infections and autoimmunity [5, 6, 7, 8]. Such defects plausibly lower the threshold for macrophage activation and cytokine amplification that define HLH. Pathogenic *KMT2D* variants disrupt epigenetic regulation of immune‐cell development, leading to impaired B‐cell maturation, altered T‐cell signaling, and reduced natural killer cell cytotoxicity, thereby predisposing patients to immune dysregulation and hyperinflammatory responses [5, 6, 7, 8].

Superimposing HLH on KS adds fever, progressive cytopenias, hepatosplenomegaly, hyperferritinemia, hypertriglyceridemia, and elevated sCD25 to a child already vulnerable to infections and nutritional stress. Importantly, marrow hemophagocytosis is not required at presentation; timely treatment is warranted once HLH‐2004 criteria (≥ 5/8) are met [3, 9]. In KS, where B‐ and T‐cell maturation is deranged and NK cytotoxicity may be reduced, autoimmune flares can serve as triggers as often as fulminant viral infections; the clinical boundary between MAS (macrophage activation syndrome) and “secondary HLH” is therefore porous [5, 6, 7, 8, 10]. This pathophysiology helps explain why immunomodulatory regimens (high‐dose glucocorticoids, calcineurin inhibition, IVIG, ± cytokine‐targeted therapy) may suffice in many KS‐HLH/MAS presentations, reserving etoposide‐based protocols for refractory or familial disease. Although the HScore was not formally calculated, several of its components, including fever, cytopenias, hyperferritinemia, and organomegaly were present, supporting a hyperinflammatory syndrome, while acknowledging its limited validation in pediatric patients [4].

HLH/MAS in KS without an infectious trigger. The negative EBV/CMV workup alongside autoimmune serologies (e.g., ANA, lupus anticoagulant) and antineutrophil antibodies supports MAS biology over infection‐driven HLH, mirroring the growing recognition of autoimmune complications in KS [5, 7, 8]. Recent literature has begun to document MAS occurring in KS, expanding the KS immune phenotype and reinforcing vigilance for hyperinflammation in KS patients with autoimmunity [10].

Diagnostic lesson. Absence of marrow hemophagocytosis despite fulfilling ≥ 5 HLH‐2004 criteria aligns with evidence that marrow findings are neither sensitive nor specific early and should not delay therapy when clinical/laboratory criteria are satisfied [3, 9].

Therapeutic responsiveness without etoposide. Clinical improvement on glucocorticoids + cyclosporine + IVIG is consistent with MAS‐oriented management strategies recommended for secondary HLH/MAS, particularly when autoimmune triggers are suspected and infection risk is high [3, 4, 11, 12]. Mortality in HLH varies by age and etiology, with higher mortality reported in adults and malignancy‐associated HLH, whereas pediatric cases, particularly those related to autoimmune or inflammatory triggers, generally demonstrate improved outcomes with early recognition and appropriate immunomodulatory therapy [9, 11].

Renal/vascular considerations in KS. Severe hypertension in a child with renal tract anomaly (e.g., ectopic kidney) is notable; calcineurin inhibitors can exacerbate hypertension and nephrotoxicity, warranting meticulous BP and renal function surveillance and a low threshold for renal imaging in KS‐HLH care plans [1].

## 5 Conclusion

This case highlights a rare association between Kabuki syndrome and HLH. The report expands the phenotypic spectrum of Kabuki syndrome and highlights the importance of early recognition of HLH in such patients, as time is very sensitive in these cases.

## Author Contributions

**Lilyan Jarrar:** data curation, writing – original draft. **Raya Fuqha:** writing – original draft. **Ahmad Mashni:** writing – original draft. **Mohammad Hamdan:** resources. **Sameer Alomari:** resources. **Mohammad Ataya:** writing – review and editing. **Yazan Abed:** writing – review and editing. **Laith Abed:** writing – review and editing.

## Funding

The authors have nothing to report.

## Ethics Statement

The authors have nothing to report.

## Consent

Informed consent was obtained from the patient's legal guardian (the parents), ensuring anonymity.

## Conflicts of Interest

The authors declare no conflicts of interest.

## Data Availability

All data generated or analyzed during this study are included in this published article. Additional data, such as laboratory values and the patient consent form, are available from the corresponding author upon reasonable request.
